# Importance of Full-Collapse Vesicle Exocytosis for Synaptic Fatigue-Resistance at Rat Fast and Slow Muscle Neuromuscular Junctions

**DOI:** 10.3390/ijms19071936

**Published:** 2018-07-02

**Authors:** Jane E. Rudling, Benjamin D. Drever, Brian Reid, Guy S. Bewick

**Affiliations:** Institute of Medical Sciences, School of Medicine, Medical Sciences & Nutrition, University of Aberdeen, Aberdeen AB25 2ZD, UK; j.e.rudling@abdn.ac.uk (J.E.R.); b.drever@abdn.ac.uk (B.D.D.); brireid@ucdavis.edu (B.R.)

**Keywords:** synaptic transmission, neuromuscular junction, myosin light chain kinase, FM1-43

## Abstract

Neurotransmitter release during trains of activity usually involves two vesicle pools (readily releasable pool, or RRP, and reserve pool, or RP) and two exocytosis mechanisms (“full-collapse” and “kiss-and-run”). However, synaptic terminals are adapted to differing patterns of use and the relationship of these factors to enabling terminals to adapt to differing transmitter release demands is not clear. We have therefore tested their contribution to a terminal’s ability to maintain release, or synaptic fatiguability in motor terminals innervating fast-twitch (fatiguable), and postural slow-twitch (fatigue-resistant) muscles. We used electrophysiological recording of neurotransmission and fluorescent dye markers of vesicle recycling to compare the effects of kinase inhibitors of varying myosin light chain kinase (MLCK) selectivity (staurosporine, wortmannin, LY294002 & ML-9) on vesicle pools, exocytosis mechanisms, and sustained neurotransmitter release, using postural-type activity train (20 Hz for 10 min) in these muscles. In both muscles, a small, rapidly depleted vesicle pool (the RRP) was inhibitor insensitive, continuing to release FM1-43, which is a marker of full-collapse exocytosis. MLCK-inhibiting kinases blocked all remaining FM1-43 loss from labelled vesicles. However, FM2-10 release only slowed, indicating continuing kiss-and-run exocytosis. Despite this, kinase inhibitors did not affect transmitter release fatiguability under normal conditions. However, augmenting release in high Ca^2+^ entirely blocked the synaptic fatigue-resistance of terminals in slow-twitch muscles. Thus, full-collapse exocytosis from most vesicles (the RP) is not essential for maintaining release during a single prolonged train. However, it becomes critical in fatigue-resistant terminals during high vesicle demand.

## 1. Introduction

Evoked neurotransmitter release occurs by exocytosis from synaptic vesicles that fuse with the terminal membrane in a Ca^2+^-dependent manner. Release comes initially from vesicles already docked at the membrane (the “readily releasable pool” or RRP) [1] but, during activity trains, other vesicles are progressively mobilized from the reserve pool (RP) [2,3]. In motor nerve terminals, most, if not all, vesicles undergo “full-collapse” exocytosis during prolonged trains, becoming an integral part of the surface membrane. This can be tracked by the release of hydrophobic vesicle markers, such as the styryl dye FM1-43 [4]. These vesicles are then recycled with an aggregate recycle time of ~110 s at ambient temperature [3,5]. Under certain conditions, however, vesicles can undergo “kiss-and-run” exocytosis, whereby neurotransmitter escapes through a small, transient fusion pore between the vesicle and terminal membranes. This pore rapidly reseals and the vesicle refills with neurotransmitter in situ. The transience of this pore opening prevents the release of FM1-43 but only partially impairs the release of the more hydrophilic dye FM2-10 (reviewed by [2]).

The actual functional significance of these two exocytosis routes and vesicle pools is an area of active research. In rat central terminals, kinase inhibitors, particularly of myosin light chain kinase (MLCK), block FM1-43 release (full-collapse exocytosis) from all but a small RRP. However, it is unknown whether this affects transmitter release or results from a switch to “kiss-and-run” or vesicle immobilization [6] or transmission failure [7]. Conversely, in frog motor nerve terminals, broad-spectrum kinase inhibitors block FM1-43 release and impair transmitter release (increase synaptic fatigue) during higher frequency trains [8,9]. However, it is not clear if these effects involve MLCK inhibition.

Much greater detail is known about the involvement of kinases, particularly MLCK, in regulating transmission at neuromuscular junctions (NMJs) of mouse semitendinosus muscle [7,10]. Here MLCK inhibition impairs FM1-43 release, and induces intermittent transmission failures during high frequency trains. Thus, MLCK activity is necessary to maintain neurotransmission at high frequency for more than a few seconds. However, this is a predominantly fast twitch muscle in which NMJs are usually only active for short intervals. Much of the muscle in mammals is slow-twitch, fatigue-resistant postural muscle where NMJs are active for prolonged periods, unlike the short duration activity bursts of fast-twitch muscle [11]. Whether kinase activity is more important for maintaining release in NMJs active for prolonged periods is unknown. We have shown that motor terminals in rat postural muscles are adapted to maintain fatigue-resistant transmission for 10 min of continuous activity, in contrast to NMJs in fast-twitch muscles. Differences in kinase regulation of transmission might exist between at least some NMJs, since Maeno-Hikichi et al. [10] found that a subpopulation of semitendinosus NMJs did not display intermittent failures, but rather underwent greater variability postsynaptic potential amplitude.

Thus, the full physiological importance of kinase activity for exocytosis mechanisms and sustained transmitter release is still not understood. In particular, their role for prolonged, low frequency activity typical of NMJs in postural muscle that makes up so much of muscle in mammals. Here, we addressed these issues by testing the effects of kinase inhibitors of varying MLCK selectivities on vesicle recycling and quantal transmitter release during postural-type physiological activity (8–10 min at 20 Hz [11] in rat motor nerve terminals with differing transmitter release fatiguabilities.

Some of these data have appeared in preliminary form [12].

## 2. Results

### 2.1. MLCK Inhibitors Block FM1-43 Release in Rat Motor Nerve Terminals

Previous studies of MLCK’s role in neuromuscular transmission focused on the frequency-dependence of vesicle supply at NMJs in predominantly fast-twitch muscle (mouse semitendinosus) [7,10]. However, the most often used NMJs are on slow/postural muscle fibers, which are adapted to maintaining release for prolonged periods (tonic activity) at low frequencies [5,10,11,13]. In mouse semitendinosus, some experiments showed two types of response to pharmacological manipulations, which may reflect such fatigue-resistant NMJs. Rats, unlike mice, have the advantage of having muscles with almost pure slow/postural-type skeletal muscles (soleus, SOL), in addition to almost pure fast/fatiguable (extensor digitorum longus, EDL). Here we tested for differences in kinase-dependence of transmitter release and synaptic vesicle recycling between such fast/phasic and slow/tonic NMJs and its involvement in adaptations to maintain release during prolonged (10 min), low frequency (20 Hz) activity. Such trains reflect the in vivo activity of fatigue-resistant terminals in soleus (SOL) [11].

**Figure 1:**
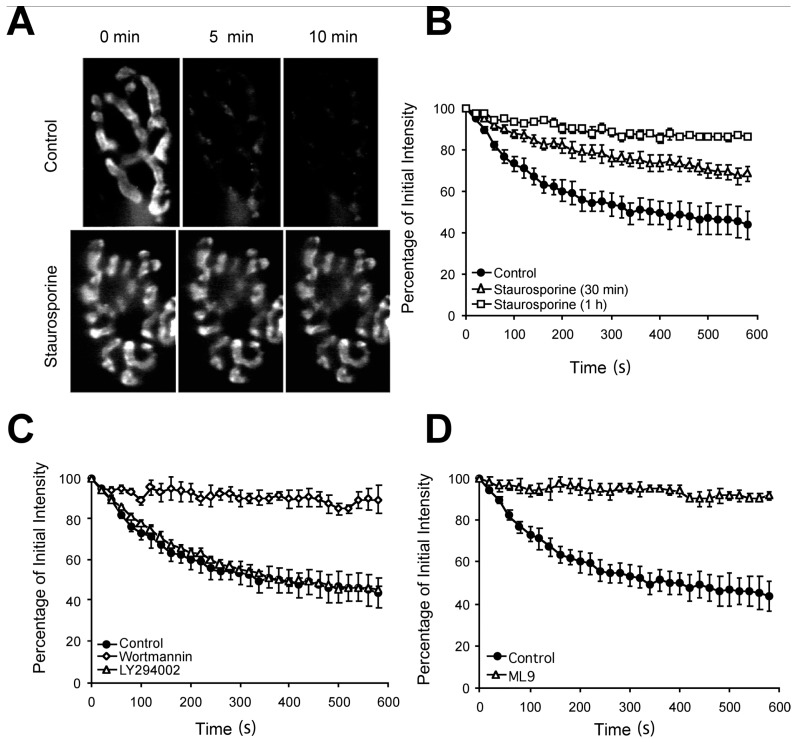
Inhibitors of myosin light chain kinase (MLCK) block activity-dependent release of FM1-43 from prelabelled motor nerve terminals of rat extensor digitorum longus (EDL) muscles. (**A**) FM1-43-labelled terminals without drug incubation (**upper panels**) or with (**lower panels**) staurosporine (2 μM, 1 h) at three time points during continuous nerve stimulation at 20 Hz. Staurosporine blocks the activity-dependent destaining seen under control conditions; (**B**) The blockade with staurosporine was maximal at 1 h incubation. The optimal incubation time for the other kinase inhibitors used in this study was determined in the same way (no drug, *n* = 6; 30 min Sta, *n* = 3; 1 h Sta, *n* = 6); (**C**) 1 μM Wortmannin (2 h incubation, *n* = 4), which inhibits both MLCK and PI3-kinase, blocks dye loss as effectively as staurosporine during 20 Hz trains. However, the PI3-kinase specific inhibitor LY294002 (1 μM, 2 h) does not affect destaining (*n* = 5); (**D**) Incubation with ML-9 (30 μM, 2 h) blocks destaining of FM1-43 labelled EDL nerve terminals during nerve stimulation (*n* = 4). ML-9 is a more selective MLCK inhibitor than stauropsorine or wortmannin. This suggests MLCK activity is required for effective FM1-43 release. No drug, *n* = 6 in all cases.

To facilitate comparison with studies in mouse semitendinosus, we began by determining the kinase pharmacology of the fast/phasic synaptically fatiguable terminals during the prolonged activity trains found in fatigue-resistant muscles. We first examined vesicle recycling modes, to ask whether like frog motor and rat central nervous system (CNS) terminals, FM1-43 loss from rat EDL motor nerve terminals is blocked by the broad-spectrum kinase inhibitor staurosporine (Figure 1A,B) [1,8,9]. Staurosporine (2 μM) profoundly inhibited the nerve stimulation-induced destaining (10 min train at 20 Hz) of FM1-43-loaded EDL terminals (Figure 1A). The block by staurosporine was time-dependent (Figure 1B), with a complete block achieved by a 1-h incubation. At this time, terminal destaining became not significantly different from dye loss in non-stimulated terminals (*p* ≥ 0.3 at 2, 5 & 10 min, *n* = 6 in each treatment). Thus, just as in frog motor and rat CNS terminals, staurosporine inhibited release of FM1-43 from preloaded rat EDL motor nerve terminals.

However, staurosporine is a broad-spectrum kinase inhibitor. Thus, these experiments do not show which kinase(s) are involved in blocking destaining. In rat CNS terminals and mouse NMJs, myosin light chain kinase (MLCK) activity is crucial for substantial FM1-43 release. We therefore compared a range of increasingly selective inhibitors of MLCK and other kinases. Wortmannin is a cell-permeant kinase inhibitor with a more restricted range than staurosporine. At low doses (<10 nM) it selectively inhibits PI3-kinase [14] but at higher concentrations it also blocks other kinases, including MLCK [15,16]. High doses (1 μM) of wortmannin (*n* = 4) inhibited FM1-43 destaining as effectively as staurosporine (Figure 1C; *p* < 0.01 vs. stimulated drug-free controls at 2, 5 & 10 min) but this was exquisitely sensitive to drug concentration. Reducing the concentration to 0.5 µM removed any detectable effect on destaining (*n* = 3; *p* > 0.5 vs. % dye loss in drug-free controls at 2, 5 & 10 min).

At 0.5 µM, wortmannin should inhibit PI3-kinase, implying PI3-kinase activity is not important for FM1-43-release-competent exocytosis. Testing this more directly, by applying the selective PI3-kinase inhibitor, 2-(4-morpholino)-8-phenyl-4H-1-benzopyran-4-one (LY294002, 1 μM, 2 h), had no effect on destaining (Figure 1C; *p* > 0.1 vs. stimulated drug-free controls at 2, 5 & 10 min, *n* = 5), confirming PI3-kinase activity is not required for FM1-43 release from rat EDL motor nerve terminals for prolonged trains.

These data implied MLCK was indeed the relevant kinase. We therefore tested this directly with the more selective MLCK inhibitor ML-9. As in previous studies, ML-9 (30 μM; 2 h, *n* = 4) entirely inhibited destaining of rat EDL terminals (Figure 1D), as completely as staurosporine or high wortmannin concentrations (*p* < 0.01 vs. stimulated drug-free controls at 2, 5 & 10 min). This suggests MLCK blockade alone is necessary and sufficient to prevent FM1-43 release during prolonged low frequency trains at rat-fatiguable NMJs.

### 2.2. MLCK Inhibitors Do Not Impair Neurotransmitter Release in EDL NMJs at 20 Hz

**Figure 2:**
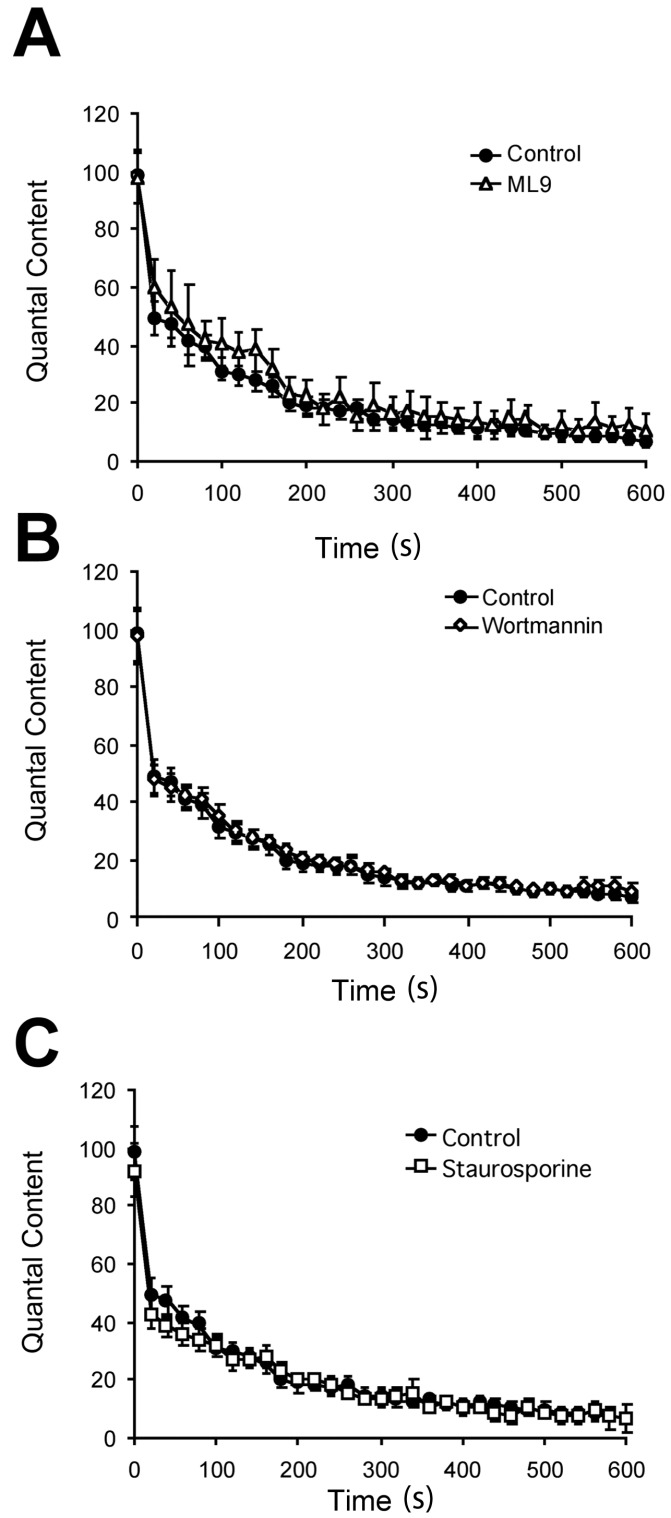
Protein kinase inhibitors have no significant effect on initial quantal content (QC) or QC rundown during 20 Hz trains. Evoked release from EDL terminals during a 20 Hz train is unaffected by either (**A**) ML-9 (30 μM, 2 h; *n* = 3); (**B**) wortmannin (1 μM, 2 h; *n* = 4); or (**C**) staurosporine (2 μM, 1 h; *n* = 8) compared with drug-free controls (*n* = 9).

Next, we tested whether this blockade of FM1-43 release impaired quantal transmitter release in these fatiguable terminals by quantal analysis, using electrophysiological recordings of postsynaptic potentials. The number of vesicles released per stimulus, the quantal content (QC), was unaffected by MLCK inhibition (ML-9, 30 μM, 2 h, *n* = 3; Figure 2A). There were no failures of transmitter release, and postsynaptic potential amplitude (mEPP and EPPs) and frequency (mEPPs) were not significantly different. Both initial QC and synaptic fatigue (QC rundown) during a train (20 Hz, 10 min; Figure 2A, *p* > 0.3 at 0, 2, 5 & 10 min) were normal (*n* = 9). Therefore, despite profound blockade of FM1-43 destaining, ML-9 did not affect sustained transmitter release at 20 Hz.

To test if blocking additional kinases would impair transmitter release, as seen in frog terminals with staurosporine [9], we examined the effects of the broader-spectrum inhibitors used in destain experiments. Interestingly, neither wortmannin (1 μM, 2 h; *n* = 4) nor staurosporine (2 μM, 1 h; *n* = 8) affected any of the transmitter release parameters measured (Figure 2B,C; *p* > 0.5 vs. drug-free controls at 0, 2, 5 & 10 min). Thus, rat EDL terminals can maintain normal levels of sustained transmitter release at 20 Hz despite a profound block of FM1-43 release.

### 2.3. A Component of FM1-43 Release Persists During Kinase Inhibition

Given such prolonged trains, it is puzzling how transmitter release can be maintained at normal levels despite the block of FM1-43 destaining, which is considered to represent full-collapse vesicle exocytosis. In rat CNS terminals [1] and mouse semitendinosus [7] MLCK inhibitors leave a small vesicle population (the RRP) still capable of undergoing full-collapse exocytosis. In rat EDL terminals, this is around 7000 vesicles (4% of total vesicle pool) from our previous studies [5]. A restricted population, if recycling very rapidly, might explain the maintenance of transmitter release. This RRP should be detectable as a small fraction of FM1-43 loss still occurring at the very start of the train. Indeed, in many cases, this is seen, on close examination of the initial destaining in Figure 1B,C, and to a lesser extent in Figure 1D.

**Figure 3:**
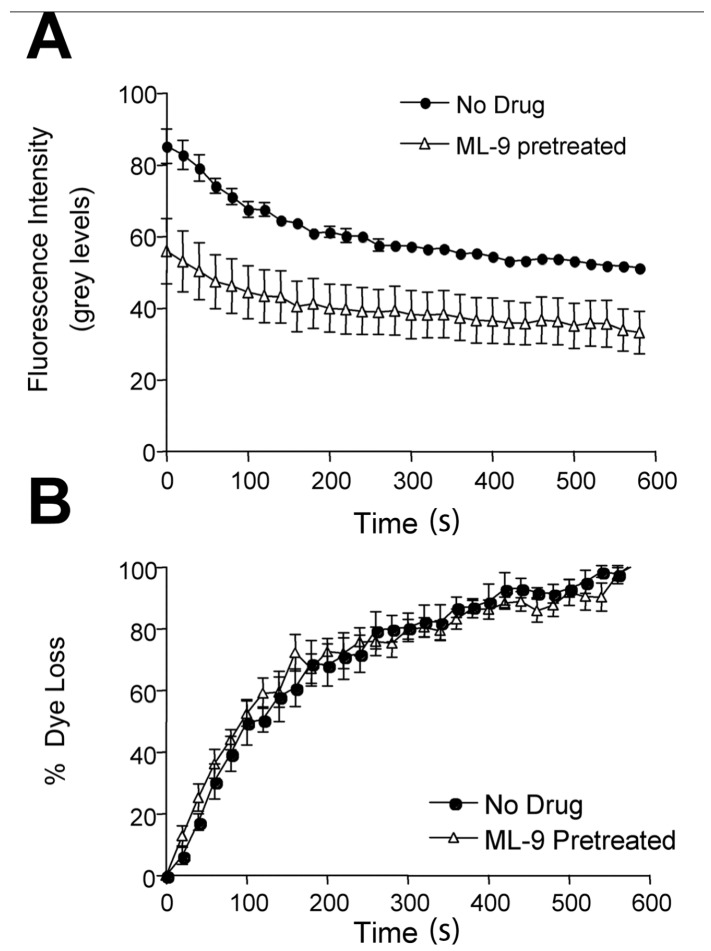
A small vesicle pool continues full-collapse exocytosis during MLCK inhibition. (**A**) Incubating in ML-9 (30 μM, 2 h) before labelling significantly reduced the activity-dependent uptake of FM1-43—i.e., initial intensity (no drug, *n* = 4 & ML-9, *n* = 7; * *p* < 0.05 vs. no drug control, Student’s *t*-test). However, this FM1-43 was freely releasable again, unlike that taken up before blockade. (**B**) Data in A, plotted as cumulative fractional dye loss per unit time to compare the timecourses of FM1-43 release. Despite the reduction in total dye uptake during kinase inhibition, those vesicles still able to undergo full-collapse exocytosis lose dye identically to drug-free terminals. Thus, during MLCK inhibition there is still a small pool of vesicles that continue to take up and release FM1-43 normally.

However, small fluorescence changes in brightly labelled terminals are difficult to quantify accurately. To test for this vesicle pool more directly and more sensitively, the normal sequence of labelling then pharmacological blockade was reversed. So, preparations were blocked first and we asked if these preblocked terminals could still internalize and release FM1-43, but in smaller quantities. In agreement with this, kinase-blocked terminals still took up FM1-43, but the fluorescence intensity was significantly less than nonblocked terminals (e.g., for ML-9 blocked terminals. *p* < 0.05; Figure 3A). Moreover, this FM1-43 was releasable again, since ML-9-blocked terminals did subsequently destain and, when normalized for intensity differences, the kinetics of dye loss was unaltered (Figure 3B). Thus, a small vesicle population continues to stain and destain in a qualitatively identical manner to nonblocked terminals—i.e., by full-collapse exo/endocytosis.

### 2.4. Estimating the Size of the Inhibitor-Resistant Vesicle Pool Releasing FM1-43

**Figure 4:**
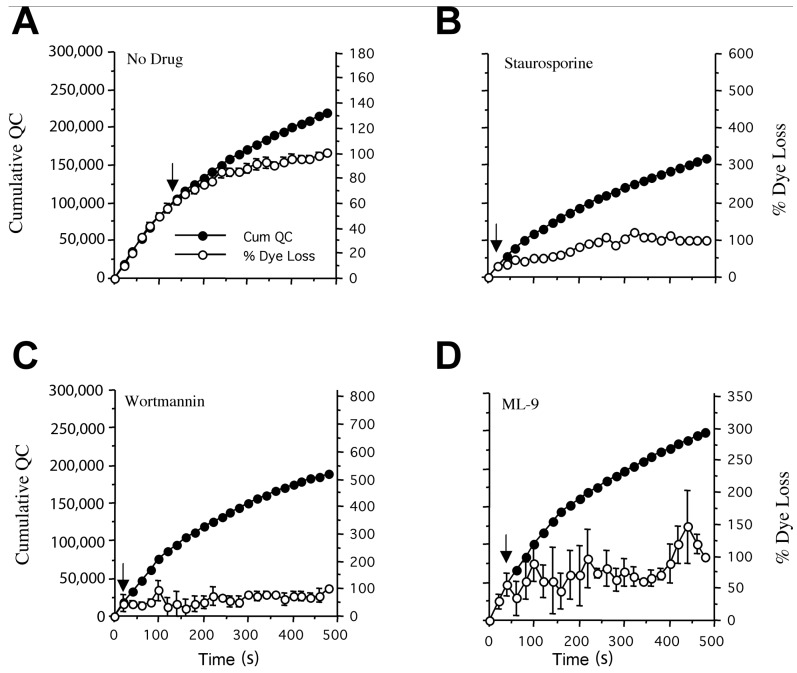
Correlating cumulative QC and FM1-43 loss to estimate the size of the inhibitor-resistant vesicle pool undergoing full-collapse exocytosis. The deviation time (arrow) for cumulative dye loss and transmitter release is 125 ± 15 s (*n* = 6) in normal terminals (**A**) compared with only 14 ± 5 s (*n* = 5), 13 ± 4 s (*n* = 3) and 30 ± 7 s (*n* = 3) after staurosporine (**B**) wortmannin (**C**) and ML-9 (**D**) treatment, respectively. Thus, kinase inhibition severely restricts the number of vesicles undergoing full-collapse exocytosis. The vesicle population releasing FM1-43 can be calculated from the cumulative QC at the deviation time (see Section 4). Overall, kinase inhibitors reduced the full-collapse exocytosis population from 154,918 ± 7625 to 18,436 ± 4211, a reduction of > 88%.

**Figure 5:**
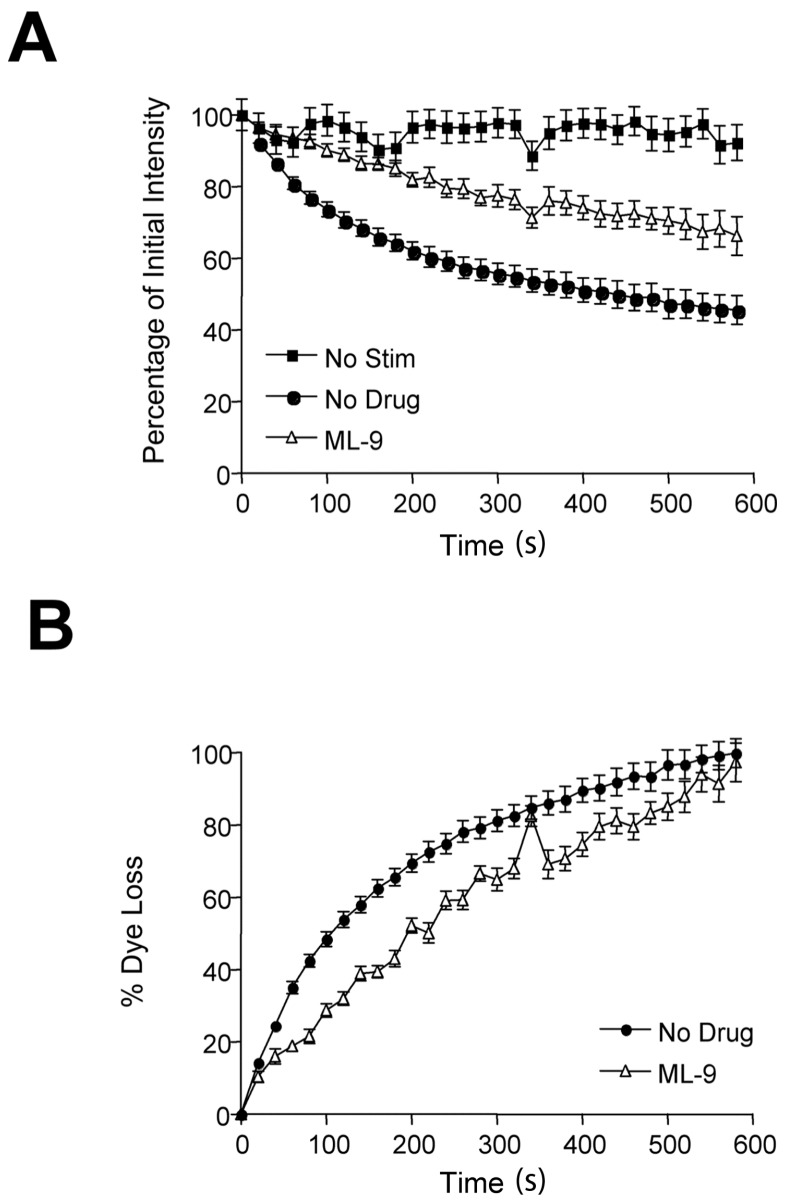
Unlike FM1-43, FM2-10 release is not completely blocked by MLCK inhibition after labelling. (**A**) FM2-10 release from drug-free, unstimulated and ML-9-blocked terminals (30 µM, 2 h). ML-9 treatment after labelling permits substantial activity-dependent FM2-10 loss, indicating kiss-and-run exocytosis is occurring. (**B**) Data in (**A**), plotted as cumulative fractional dye loss per unit time. During MLCK inhibition, the initial FM2-10 release rate is normal, probably reflecting the ongoing full-collapse exocytosis from the earliest vesicles. However, the later pattern is more linear, consistent with an altered exocytosis mode from the later vesicles (no drug, *n* = 7 & ML-9, *n* = 8).

To estimate the size of this vesicle population and compare it to previous electrophysiological measures of RRP size in these terminals, destaining pool size estimates were determined by correlating the cumulative dye loss with the number of quanta released during the stimulation train [5]. For FM1-43 release, dye loss and transmitter release correlate well initially in unblocked terminals, as each vesicle releases both FM1-43 and transmitter simultaneously. At later times, however, recycled vesicles that are depleted of dye start to contribute, slowing dye loss relative to transmitter release. This lag (i.e., vesicle recycle time) is typically 110–120 s (Figure 4A and [5]). The cumulative quantal release shows the number of vesicles involved in FM1-43 loss (see Methods and [5,13]). In blocked preparations, dye loss was normal initially, but then quickly lagged behind transmitter release—typically within 20–30 s (second or third image intensity data point in Figure 4B–D). Mean data for the best-fit analysis are given in Table 1. The mean time to the deviation point was 18 ± 3 s (*n* = 11); substantially shorter than for drug-free terminals (125 ± 15 s, *n* = 6; *p* < 0.005) [5,13]. This corresponds to a mean kinase inhibitor-resistant FM1-43 destaining pool size of 18,436 ± 4211 (*n* = 11), or 11.9% of the total vesicle pool of 154,918 ± 7625 in controls, with a recycle time of ~110 s reduced to 18 s. Staurosporine and wortmannin yield similar estimates (11,000), which agree more closely with our previous electrophysiological estimates of the RRP (7000). Higher estimates obtained with ML-9, the more selective MLCK inhibitor may simply reflect the greater noise in the data. Or, this may indicate that this more selective kinase inhibitor is uncovering a third, intermediate pool of vesicles. From the data mean, this still indicates that in the presence of MLCK inhibitors, the number of vesicles undergoing FM1-43 releasing exocytosis is around 12% of normal ((18,436/154,918) × 100).

### 2.5. MLCK Inhibition Slows, but Does Not Block, FM 2–10 Release

The persistence of this small, rapidly recycling population of vesicles able to take up and release of FM1-43 suggests at least some transmitter release during kinase inhibition involves full-collapse exocytosis. However, transmitter exocytosis can also occur by other mechanisms, particularly kiss-and-run. This is detectable using a related styryl dye, FM2-10, which is more hydrophilic and has faster membrane washout kinetics [17] allowing some release during kiss-and-run exocytosis [18]).

In nonblocked terminals, FM2-10 and FM1-43 uptake and release were identical (c.f. Figure 1B–D and Figure 4B). In ML-9 blocked terminals, unlike FM1-43, FM2-10 still underwent substantive release (Figure 5A). However, the rate of dye loss was markedly reduced (c.f. Figure 1), with 50% dye loss taking ~200 s (Figure 5B). Notably, destaining rate during the first 10 s was normal, consistent with the early component of ML-9-resistant destain.

The slow, linear pattern of FM2-10 destaining induced by ML-9 treatment does not simply reflect wash-out of dye sticking to surface membranes. First, the background subtraction algorithm should remove this artefact. Second, unstimulated terminals hardly destained (Figure 4A, “No Stim”). And, third, experiments removing noninternalized dye with Advasep-7 during the wash and ML-9 incubation produced a similar outcome, just lowered the initial background intensity (data not shown). Advasep-7 is a cell-impermeant FM dye chelator [19], which removes FM dyes from exposed membranes. While it reduced background labelling intensity.

Therefore, these observations indicate that MLCK inhibition leaves two exocytosis routes available for transmitter release: a minor inhibitor-resistant component that permits FM1-43 release, and a much large component which permits FM2-10 release, but not FM1-43. Thus, the ability of blocked terminals to maintain normal transmitter release during prolonged 20 Hz trains reflects continuing exocytosis from a large whole vesicle pool, but by at least two separable mechanisms.

### 2.6. Sustained Transmitter Release from Fatigue-Resistant Terminals Is More Sensitive to Kinase Inhibition

Overall, therefore, MLCK activity is not essential for maintaining neurotransmission in these NMJs, despite controlling recycling mechanisms in most of the vesicle population. Perhaps increasing release demand would reveal synaptic impairments. A standard means to do this is to increase stimulation frequency, particularly in vivo frequencies (~100 Hz). However, this results in essentially normal release events alternating with complete failures by an unknown mechanism [7,10], suggesting that more complex effects dominate under these conditions. We therefore increased vesicle release using elevated extracellular Ca^2+^ (5 mM), enhancing release probability. To compare the effects of the elevated Ca^2+^ manipulations directly, release in drug-free and kinase-inhibited preparations were both normalized to normal (2 mM) Ca^2+^, in drug-free conditions. Moreover, to ensure maximum kinase inhibition, and hence minimising FM1-43-competent recycling pool size, we used broad-spectrum inhibitors staurosporine or wortmannin.

**Figure 6:**
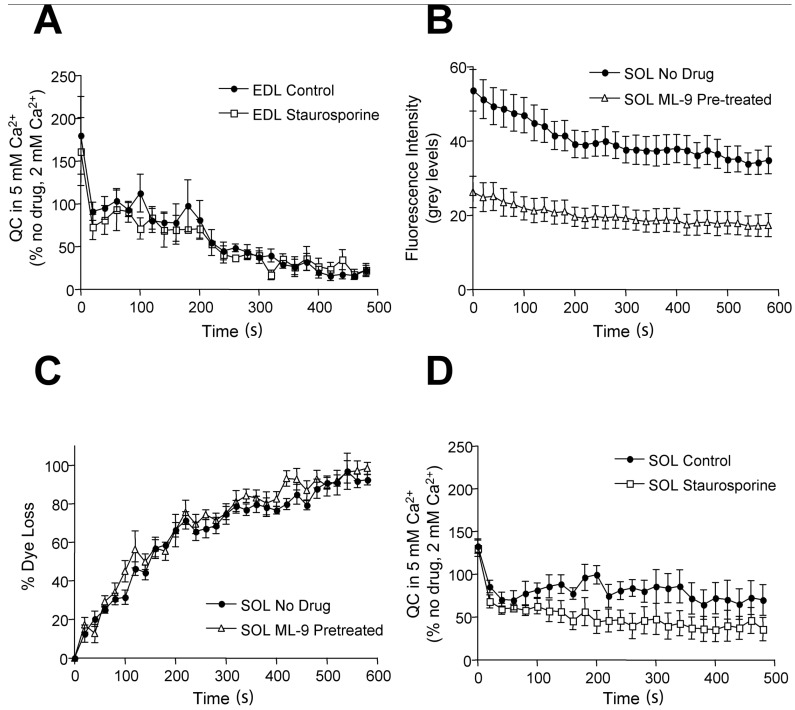
The effects of kinase inhibitors on sustained transmitter release when QC is augmented by high (5 mM) extracellular Ca^2+^ in fatiguable (EDL) and fatigue-resistant (SOL) terminals. (**A**) Plot of QC_hi Ca_/QC_lo Ca_ during 20 Hz trains for EDL terminals, expressed as a percentage. High Ca^2+^ enhanced initial QC and increased QC rundown. The impairment (i.e., <100%) is particularly evident from 200 s. However, staurosporine does not exacerbate this rundown (no-drug control, *n* = 4 & 2 µM staurosporine, *n* = 3); (**B**) SOL terminals, like EDL, have fewer vesicles undergoing full-collapse exo- and endocytosis in MLCK inhibitors. As for EDL, MLCK (30 μM ML-9, 2 h; *n* = 6) reduces FM1-43 uptake, i.e., initial intensity, compared with no-drug control SOL terminals (*n* = 5). The small amount of FM1-43 internalized, however, is releasable; (**C**) The cumulative dye loss for the data in B shows that, like EDL, the fractional rate of FM1-43 dye release is the same as from drug-free terminals despite the reduction in population size. This indicates a small population continues full-collapse exocytosis during MLCK inhibition; (**D**) staurosporine impairs QC rundown in SOL terminals augmented by 5 mM Ca^2+^. 5 mM Ca^2+^ increases initial QC in both no-drug and staurosporine-treated terminals. However, staurosporine significantly increases QC rundown during a 20 Hz train (*n* = 6) compared with no-drug SOL terminals (*n* = 5; two-way ANOVA, *p* < 0.0001), making them not significantly different from corresponding EDL terminals (two-way ANOVA, *p* > 0.25).

As expected, in drug-free conditions, higher extracellular Ca^2+^ substantially enhanced the number of vesicles released for the first EPP in the train (initial QC). Subsequently, QC rundown (synaptic fatigue) was increased (Figure 6A), but in a biphasic manner. QC first decreased quite quickly to control (2 mM Ca^2+^) levels. This was well maintained until ~200 s, after which synaptic fatigue was enhanced, falling to only ~10% of that in normal 2 mM Ca^2+^ by the end of the train. Surprisingly, neither wortmannin nor staurosporine enhanced synaptic fatigue. Thus, 5 mM Ca^2+^ increased the initial QC in drug-free (134.2 ± 33.9, *n* = 4 vs. 85.2 ± 16.0 in 2 mM; *p* < 0.05) and staurosporine-treated (120.1 ± 36.3, *n* = 4, 5 mM vs. 89.1 ± 12.0, 2 mM; *p* < 0.05) terminals equally (ANOVA, no significant difference between no drug & staurosporine), as well as QC rundown (*n* = 5; QC non-blocked vs. staurosporine at 2, 5 & 8 min). Synaptic vesicle refilling was also unaffected, since mEPP amplitudes before and after these prolonged trains were the same (e.g., in staurosporine, pre-train mEPP amplitude 0.36 ± 0.05 mV vs. 0.33 ± 0.06 mV immediately after; 30 mEPPs/fiber, *n* = 3–6 fibers each; NS paired *t*-test). Thus, even under conditions of increased QC, altering vesicle exocytosis mode had no effect on EDL terminals’ ability to maintain normal release at 20 Hz.

Under these drug-free conditions, QC in EDL terminals typically decreases to ~10% of initial values in normal saline (Figure 2) [5]. Thus, it may simply be difficult to detect an enhancement of an already large synaptic fatigue. Nonetheless, these experiments indicate that during a prolonged train at low frequency, kinase activation is not functionally important in fatiguable synapses. We therefore next asked whether extensive full-collapse vesicle recycling was more important for terminals that normally maintain high levels of release for prolonged periods—i.e., fatigue-resistant synapses. In the postural SOL muscle, terminals in vivo maintain release for several minutes at a continuous 20 Hz, compared with EDL (intermittent 1–2 s bursts at ~100 Hz) [11]. This is because SOL terminals have a smaller initial QC (~85), and a larger RRP (~8500) and total vesicle pool (~250,000) than those in EDL (~100, ~7000 and ~178,000, respectively [5]).

In drug-free, 2 mM Ca^2+^ conditions, as shown previously [5], initial QC was smaller than in EDL, and QC rundown was very much slower (two-way ANOVA, *F* = 19.81, *p* < 0.0001; SOL *n* = 5 & EDL *n* = 4). During kinase inhibition of SOL terminals, most changes were very similar to those seen in EDL, so only indicative data are presented here (Figure 6B–D). The release and uptake of FM1-43 was decreased to a similar extent (c.f. Figure 3 for EDL), but transmitter release and synaptic fatigue were unaffected. In 5 mM Ca^2+^, again like EDL NMJs, initial QC was increased similarly in staurosporine-treated and drug-free preparations. However, kinase inhibition in 5 mM Ca^2+^ induced a significant enhancement of synaptic fatigue in SOL NMJs (two-way ANOVA, effect of drug, F = 58.71; *p* < 0.0001; staurosporine *n* = 5, no drug *n* = 6). Indeed, it entirely removed the synaptic fatigue resistance in SOL compared with EDL (two-way ANOVA, F = 1.09, *p* > 0.25; SOL *n* = 6 & EDL *n* = 3). Thus, under conditions of augmented release, kinase activity is responsible for the differences in synaptic fatiguability between EDL and SOL NMJs. This suggests kinase-dependent, FM1-43-competent vesicle exocytosis is an adaptation to maintain release under sustained conditions of high volume vesicle exocytosis.

## 3. Discussion

The evidence for various means of neurotransmitter exocytosis (full-collapse, kiss-and-run, kiss-and-stay) is well established, but their physiological significance remains unclear. In NMJs undergoing long continuous activity, full-collapse vesicle recycling (defined as supporting full FM1-43 uptake/release) is the default mode for transmitter release for most of the vesicle pool. The present study investigated whether FM1-43-releasing exocytosis is necessary for sustained transmitter release, by comparing terminals physiologically adapted for different synaptic fatiguabilities.

For comparison with previous studies of short, high-frequency activity trains typical of fast-twitch fatiguable muscles [7,10,20], we first examined sustained low-frequency firing in NMJs in fatiguable rat EDL in which transmitter release falls by >80% of initial QC during prolonged 20 Hz trains. Neurotransmitter secretion in fatiguable EDL NMJs predominantly involved FM1-43 release, the majority of which was kinase-, apparently MLCK-, dependent. However, while kinase inhibition spared a small vesicle population which continued full collapse recycling, the majority of transmitter is via a mode that releases FM2-10, but not FM1-43, even when release was artificially elevated with increased extracellular Ca^2+^. Essentially identical observations were made in SOL NMJs in normal release conditions of 2 mM Ca^2+^. Thus, FM1-43-releasing exocytosis also seemed largely redundant for sustaining much higher transmitter release for the long stimulation trains in SOL NMJs. However, kinase inhibition entirely blocked the synaptic fatigue-resistance element of release during elevated demand (5 mM Ca^2+^), suggesting this mode of recycling is required to sustain high volume release.

The present studies extend earlier observations in rat CNS and frog terminals [1,9], that a range of kinase inhibitors block FM1-43 release from most synaptic vesicles, to include rat motor nerve terminals. Similarly, there is a small population of vesicles resistant to such inhibitors that continue using this mode. This mode of release is believed to indicate full-collapse vesicle recycling [1,7,10]. The majority now use a mode that releases FM2-10, but not FM1-43, regarded as a marker of kiss-and-run exocytosis.

It is possible the characteristic block of most FM1-43 release is an artefact of the experimental conditions, particularly of phototoxicity from FM dye illumination, perhaps enhanced by kinase inhibition. However, this seems unlikely for a number of reasons. First, only inhibitors of MLCK produce this effect. Second, we use extremely low light levels (always <4% of maximum, and usually <1.6%), which in previous control experiments allowed imaging of at least two consecutive rounds of FM1-43 loading and unloading in the absence of inhibitors [5,21]. Third, release of the closely chemically related FM2-10 is much less disturbed in the presence of the same inhibitors, despite being present at 50× higher concentration. And, finally, in the absence of illumination (Figure 4), MLCK inhibitors alone prevent FM1-43 uptake by most vesicles. Thus, it seems only inhibitors of MCLK can produce these effects, and they specifically affect the FM1-43 uptake/releasing, full collapse exocytosis.

Of the kinase inhibitors tested, only those that inhibit MLCK block FM1-43 release, implying MLCK activity is necessary for full-collapse exocytosis from most vesicles under these conditions. While previous studies all agree MLCK plays a pivotal role in regulating routes of vesicle recycling, our specific observation contrasts directly with that in fast-twitch mouse semitendinosus [10], where kiss-and-run exocytosis is induced by activation, not inhibition, of MLCK. The reason for the contrasting results is unclear, but there are three important differences in the two studies. First, the species. While it is possible there are rat/mouse differences in regulation of exocytosis routes, this seems unlikely. Second, the frequency of activation. In mice, frequency of stimulation clearly modulates exocytosis routes. It may be the exocytosis route supported by kinase activity depends on frequency. Alternatively, MLCK activity may have reciprocal effects on exocytosis routes in the two vesicle pools—with the RRP using kiss and run during kinase activation, which when dye-depleted, still release transmitter but impair access to release sites by mobilized RP vesicles using full collapse. This would slow dye loss but not transmitter release. The converse situation at low frequency, could explain findings in the current study. Finally, duration of activity trains. In the mouse, predicated on the opposite physiological activity pattern, the focus was on a few seconds of activity, with even the longest trains lasting only 100 s (10,000 stimuli @ 100 Hz). In the present study, with stimulation trains of 10 min, and imaging every 10 s, the temporal resolution to plot changes accurately at these very early times was lacking. Rather, the current focus was on the ability to maintain release during fatiguing trains. Further studies are therefore required to test how MLCK, perhaps with other kinases, regulates the modes of vesicle exocytosis, and whether it may change depending upon timescale and frequency of activation.

For the present experiments, fatigue of transmitter release was unaffected during long low frequency trains typical of rat postural muscles, even with broad spectrum inhibitors, showing a robust mechanism for maintaining release is present. However, it did not have an absolute requirement for full-collapse exocytosis. Most vesicles switched to kiss-and-run during kinase inhibition, releasing FM2-10, but not FM1-43. Thus, release was maintained, but was adaptable to the mode of exocytosis.

While release from EDL was maintained at normal levels, these synapses normally fatigue quite readily, reflecting their adaptation to short, high frequency activation in vivo. They might be expected, therefore, to show enhanced fatigue when full collapse vesicle exocytosis is restricted. However, even when release was increased in high extracellular Ca^2+^, this was not the case. This, together with the restricted number of vesicles in the pool in this type of terminal, determined from previous studies in our laboratory [5,13], suggests the fatigue in neurotransmitter release here reflects an inadequate supply of vesicles, rather than an inadequacy of the mode of exocytosis. This conclusion is reinforced by the observation that SOL terminals, which have a much larger vesicle pool, were also able to maintain a much higher level of release during the same activity patterns. It was only when release was excessively high, boosted in this case by elevated extracellular Ca^2+^, that SOL terminals were no longer able to maintain neurotransmitter release during kinase inhibition. This implies, therefore, that full-collapse vesicles exocytosis is better able to support high volume release during periods of high vesicle demand.

### 3.1. Kinase Inhibitors

The inhibitors chosen were all used previously in similar studies. While none of the kinases are specific for MLCK, only those which do block MLCK affected the ability to release FM1-43. This correlation between MLCK inhibition and the ability to block FM1-43 release supports previous studies implicating MLCK in regulating exocytosis modes. In contrast to frog and mouse terminals, these inhibitors did not impair transmitter release here. This probably relates to the low frequency stimulation used in the current study—a paradigm based on SOL’s in vivo activity patterns. Moreover, the intermittent complete failures of transmission at mouse NMJs during high frequency stimulation may indicate periodic axonal block of action potential propagation. Prolonged high frequency trains in drug-free, fast-twitch EDL can induce intermittent transmission failure of this sort [5]. This increased incidence may indicate another role for this clearly important kinase.

In the present study, high wortmannin concentrations (1 μM), like staurosporine, inhibited FM1-43 loss without impairing maintained transmitter release. This is similar to findings in frogs [22], where wortmannin only reduced sustained release on repeated trains of stimulation. This was attributed to inhibition of endocytosis, which is PI3-kinase-dependent [23]. Wortmannin’s effects are distinctly concentration dependent. At much higher concentrations (10–100 μM) wortmannin can impair evoked release from rodent motor nerve terminals [24], whereas at lower concentrations in the current study (0.5 µM), release of both transmitter and FM1-43 were both normal. In hippocampal neurone cultures [25], 10 µM wortmannin did not block FM1-43 release. The reason for this is not entirely clear, but perhaps reflects the much shorter incubation times used (15 min). Rather, two hours was required in the present study for maximum blockade of FM1-43 release. Alternatively, as they suggested, larger terminals may be more dependent on the myosin-actin system.

The PI3-kinase inhibitor LY294002 did not affect either dye loss or transmitter release. We deliberately used much lower concentrations (1 µM) in the current study than those reported to impair transmitter release in other preparations [22,24]. In frogs, 200-μM LY294002 impaired vesicle recycling by its inhibition of casein kinase-2 at this concentration, rather than by its effects on PI3-kinase. It may be that these lower concentrations may not fully inhibited PI3-kinase (IC_50_ 1.4 μM [26]). However, wortmannin blocks PI3-kinase at low nanomolar concentrations [27], yet even 0.5 μM wortmannin in the present study did not affect either FM1-43 or transmitter release. We conclude, therefore, that PI3-kinase activity is not essential for supporting FM1-43 release/full-collapse exocytosis or transmitter release in rat motor nerve terminals during a single train at modest frequencies.

The MLCK inhibitors ML-9 and its analogue ML-7 can reduce Ca currents, action potential firing or induce intermittent neurotransmission [7,25] as well as inhibit vesicles movements [6]. Since QC was normal and there was no intermittent firing in the present study, the block of FM1-43 release was independent of these side-effects.

### 3.2. Vesicle Exocytosis in Kinase-Blocked Terminals

For each inhibitor, the effectiveness of FM1-43 destain-blockade correlated directly with its ability to inhibit MLCK. During such blockade, dye release patterns indicated two distinct vesicle pools. A small vesicle population continued to take up and release FM1-43, indicating ongoing full-collapse exocytosis. This accords with previous findings in CNS terminals [1], plus the endocytic internalisation of macromolecules such as synaptotagmin antibodies into some vesicles in staurosporine-blocked terminals [28].

This MLCK-resistant population seems likely to reflect the RRP. Functionally, it is a small, early pool of vesicles still able to release FM1-43 during MLCK inhibition [1]. It is also similar in size to previous electrophysiological measures of the RRP in nonblocked terminals [5]. In frogs, it corresponds to a population near the active zone that is selectively depleted in staurosporine-treated terminals stimulated at higher frequencies [9].

With respect to the much larger kinase inhibitor-sensitive vesicle population, our data indicate an essentially exclusive move to kiss-and-run exocytosis. First, staurosporine induces reduction of exo- and endocytosis time [8,28], and total recycle time (present study—from ~110 s to ~20 s, Figure 4). Second, the extensive release of FM2-10 (~50% of unblocked terminals; Figure 4) shows at least most of these vesicles continue to undergo some form of exocytosis. Moreover, the electrophysiological recordings show transmitter release from kinase-blocked terminals is quantal, i.e., vesicular, in nature. Thus, these data show substantial exocytosis occurs, at normal levels, but by an altered mechanism. How this observation relates to reports of vesicle immobilisation by staurosporine [1,8,28] or other MLCK inhibitors [6] is uncertain. For example, it is unclear how an immobilized vesicle population can reach the terminal membrane to release transmitter and FM2-10. Kiss-and-run exocytosis, therefore, seems the most appropriate functional description of the larger vesicle pool recycling during blockade with MLCK inhibitors.

Full-collapse exocytosis is clearly the default exocytosis mechanism for the vast majority of vesicles in these presynaptic terminals, since drug-free terminals readily internalize and release substantial amounts of FM1-43. Equally, however, electrophysiology shows it is not essential to maintain normal transmitter release at modest frequencies found in vivo. This raises the question then of general principles—when is full-collapse exocytosis from the full vesicle pool functionally essential? That was the question at the core of the current study.

Kinase inhibition-induced impairment of transmitter release seems to correlate with a high vesicle/transmitter demand. Frog terminals have a very high initial QC (~200 [29] and MLCK inhibitors impair transmitter release during higher frequency trains. While EDL terminals also have a high initial QC (80–100 [5,30]), this is not sustained even in the absence of inhibitors. Finally, while SOL terminals have a slightly smaller initial QC than EDL (70–85 [5,30]), but normally this is well maintained. The present study shows kinase inhibition removes this synaptic fatigue-resistance adaptation if vesicle demand is increased. Thus, full-collapse exocytosis from the major vesicle pool seems particularly well suited to supplying large numbers of vesicles during sustained demand, and studies to date all indicate MLCK is a key enzyme for regulating the availability of vesicles able to undergo this mode of exocytosis.

## 4. Methods

### 4.1. Dissection

Mature (>350 g) 12-week-old male Sprague-Dawley rats were killed by legally stipulated methods (Schedule I, Animals (Scientific Procedures) Act, 1986 of the UK government). No in vivo work was involved. However, all work took place on licensed premises and complied with ARRIVE, national and institutional regulations for the ethical treatment of animals. Extensor digitorum longus (EDL) and soleus (SOL) nerve-muscle preparations were removed, mounted in silicon rubber (Sylgard, Dow Corning, Germany) -lined dishes and bathed in gassed (95% O_2_, 5% CO_2_) saline [31]. The saline was of the following composition (in mM): NaCl 138.8; KCl 4; NaHCO_3_ 12; KH_2_PO_4_ 1; MgCl_2_ 1; CaCl_2_ 2 and glucose 11, pH 7.4. All experiments were performed at room temperature (18–22 °C).

### 4.2. Styryl Dye Labelling and Imaging

Nerve-muscle preparations were soaked for 15 min in either 1 μM FM1-43 or 50 μM FM 2-10 (Molecular Probes, Leiden, The Netherlands) dissolved in gassed saline to allow good dye penetration. The nerve was then taken up into a suction electrode and synaptic vesicles were labelled by nerve stimulation in styryl dye solution. The stimulation regime for labelling was alternating trains of 10 Hz (10 s) and 1 Hz (5 s) for 15 min. Previous experiments have shown that this paradigm fully labels the terminals, and hence all recycling vesicles. Thus, longer or different paradigms of stimulation did not increase the fluorescence intensity [5]. The stimulation pulses were applied via an AMPI Master-8 pulse generator and an AMPI Iso-flex amplifier/stimulus isolator (Intracel Ltd., Issaquah, WA, USA). The preparations remained in dye solution for a further 5 min to allow endocytosis to reach completion and were then washed in gassed, dye-free saline for 30–120 min (time-matched in experimental and controls) to remove dye that had partitioned into external membranes. To prevent contraction during imaging, muscles were paralysed by exposure to 5 μM d-tubocurarine during the last 30 min of the wash to block the binding of acetylcholine to postsynaptic receptors. To determine the effects of the protein kinase inhibitors, preparations were incubated for up to 1 h in staurosporine (2 μM), or for 2 h in wortmannin (0.5–1 μM), LY294002 (1 μM) or ML-9 (30 μM) before (Figure 3 and Figure 6) or after (Figure 1, Figure 2, Figure 4, Figure 5 and Figure 6) terminal labelling. Once added, all kinase inhibitors were present throughout the rest of the experiment. Again, drug-free controls were time-matched for comparison.

To monitor exocytosis optically, images were acquired at 10 s intervals from a single terminal per muscle during stimulation. Images were acquired using a cooled charge-coupled device camera (Mono Coolview; PhotonicScience, East Sussex, UK) attached to an M2B microscope (Micro Instruments Ltd., Oxford, UK). Terminals were viewed through a Zeiss (Oberkochen, Germany) 40× water immersion objective (0.75 N.A.) and illuminated with a Prior Scientific (Cambridge, UK) HB100 mercury arc light source through a standard FITC filter set (B2A; Nikon, Surrey, UK). To minimise photobleaching and phototoxicity, a suitable *en face* superficial terminal was located quickly using light attenuated by neutral density filters to, at most, 3.13% of full lamp intensity. Thereafter, fluorescent illumination occurred only during image capture. Illumination times were controlled via a Uniblitz D122 shutter driver (Vincent Associates, Rochester, NY, USA) in the light path. During image acquisition, light intensity was further attenuated by neutral density filters to 1.56 or 0.78% of maximum intensity. OpenLab software (Perkin Elmer Ltd., Seer Green, UK) controlled the shutter opening times and camera (exposures were 8.4 s), and images were saved onto an Apple PowerMac computer.

### 4.3. Intracellular Recording

Excised muscles were bathed in 2 μM μ-conotoxin GIIIB in gassed saline, until no contraction was present (60–120 min). At this concentration, the toxin blocks the muscle action potential by preferentially inhibiting the muscle voltage-gated sodium channels [32], while normal spontaneous and nerve-evoked transmitter release can be recorded postsynaptically [5,33]. For kinase-blocking experiments, 2 μM staurosporine, 0.5–1 μM wortmannin or 30 μM ML-9 were included with the toxin solution. Standard electrophysiological techniques were used to record miniature end plate potentials (mEPPs) and evoked EPPs, during stimulation of the muscle at 20 Hz for 10 min, using glass microelectrodes filled with 3 M KCl. Initial resting membrane potentials were more negative than −65 mV and, after a small initial fall, were stable during the 10 min of the experiment and always greater than −50 mV. Potentials were recorded via an Axoclamp 2B amplifier (Axon Instruments, Foster City, CA, USA) and stored simultaneously on a digital tape recorder (Bio Logic DTR-1204; Intracel Ltd.) and the hard drive of a Walters 286 personal computer running Strathclyde Electrophysiology Software Whole Cell Program (WinWCP; John Dempster, Strathclyde Electrophysiology Software, University of Strathclyde, Glasgow, UK).

### 4.4. Data Analysis

Data analysis was predominantly by methods described previously (Reid et al., 1999). Image analysis of terminal destaining was carried out using NIH Image (available online: http://rsb.info.nih.gov/nih-image/). Briefly, all fluorescent regions of a terminal that were in focus were selected manually for measurement of pixel intensity. A background region on adjacent muscle fibers was also selected and the terminals intensities were background-subtracted to give a “net intensity” value above muscle fiber labelling and to cancel out background variability between terminals. The data for all the images of a time series were compiled and analyzed using Microsoft Excel. To compare the extent of destain between different terminals, the net intensity data were expressed as a percentage of the brightness of the start image with respect to background (nearby, nonterminal, muscle fiber region). As the start image is taken prior to stimulation of the terminal, the dye present in a terminal at the start of the experiment is defined as 100%, while the background intensity is defined as 0%. Referencing all destains to a nonterminal region in this way allows stimulated dye loss to be compared between terminals undergoing comparable experimental procedures.

Electrophysiological data were analyzed using WinWCP (John Dempster, Strathclyde Electrophysiology Software, University of Strathclyde, Glasgow, UK). Quantal content (QC) was determined by the direct method (i.e., the ratio of the mean EPP amplitude to the mean MEPP amplitude) after correcting EPP amplitude for nonlinear summation, using the formula:

QC = E ÷ (M × (1 − *f*(E ÷ Vm))) (1)

where, E is mean EPP amplitude, M is mean mEPP amplitude and *f* = 0.8 [30,33,34]. Reversal potential was assumed to be 0 mV.

To determine the size of the vesicle pool undergoing full-collapse exocytosis, FM1-43 loss and transmitter release during trains were plotted cumulatively and superimposed. Net intensity (NI) data were converted into cumulative dye loss, as a percentage. Dye loss before stimulation commenced was 0% and at the end of stimulation was 100%. Thus, the percentage dye lost after *n* images is:

[(NI_0_ − NI_n_) ÷ (NI_0_ − NI_end_)] × 100 (2)

where, “0” and “end” are the first and last images, respectively. Full-collapse vesicle pool population size was determined by superimposing cumulative dye loss and QC data at time 0, then scaling the FM1-43 loss curve to fit the cumulative QC data for as long as possible [4]. The dye loss invariably fell below the cumulative QC plot within 3 min as recycled vesicles, now devoid of dye, were released a second time. These dye-depleted vesicles produce an electrical event but no dye loss. The deviation time (DT) was determined by eye as the minimum time at which divergence of the two signals occurred. This method has been demonstrated to be as accurate as mathematical best-fit prediction [21].

FM1-43-releasing vesicle pool size was determined at DT as:

(100 ÷ % FM1-43 loss at DT) × cumulative QC at DT(3)

Thus, if 100,000 vesicles at DT equates to 65% of FM1-43 loss, vesicle pool size would be (100/65) × 100,000 = 153,846 vesicles.

### 4.5. Statistics

Data are given as mean ± standard error of the mean. The statistical significance of differences between means was tested using Student’s *t*-test for samples of equal or unequal variances, according to a prior *F*-test. Two-way analysis of variance (ANOVA) was used to test for differences in transmitter rundown patterns. For all analyses, a probability of *p* < 0.05 was taken as significant.

## Figures and Tables

**Table 1:** Destaining pool size for FM1-43 labelled rat EDL terminals.

Drug	n	Deviation Time (s)	Destaining Pool Size
No Drug	6	125 ± 15	154,918 ± 7625
Staurosporine	5	14 ± 5 *	11,250 ± 3049 **
Wortmannin	3	13 ± 4 *	12,257 ± 3192 **
ML-9	3	30 ± 7 *	36,592 ± 7451 **

* *p* < 0.005 compared with corresponding no drug control. ** *p* < 0.0001 compared with corresponding no drug control.
